# Surrogate utility estimation by long-term partners and unfamiliar dyads

**DOI:** 10.3389/fpsyg.2015.00315

**Published:** 2015-03-25

**Authors:** Richard J. Tunney, Fenja V. Ziegler

**Affiliations:** ^1^School of Psychology, University of NottinghamNottingham, UK; ^2^School of Psychology, University of LincolnLincoln, UK

**Keywords:** surrogate decisions, surrogate accuracy, utility estimations, decision-making, perspective-taking

## Abstract

To what extent are people able to make predictions about other people's preferences and values?We report two experiments that present a novel method assessing some of the basic processes in surrogate decision-making, namely surrogate-utility estimation. In each experiment participants formed dyads who were asked to assign utilities to health related items and commodity items, and to predict their partner's utility judgments for the same items. In experiment one we showed that older adults in long-term relationships were able to accurately predict their partner's wishes. In experiment two we showed that younger adults who were relatively unfamiliar with one another were also able to predict other people's wishes. Crucially we demonstrated that these judgments were accurate even after partialling out each participant's own preferences indicating that in order to make surrogate utility estimations people engage in perspective-taking rather than simple anchoring and adjustment, suggesting that utility estimation is not the cause of inaccuracy in surrogate decision-making. The data and implications are discussed with respect to theories of surrogate decision-making.

## Introduction

Making decisions can be very difficult and stressful, particularly when they have significant implications for our health, finances and future happiness. As we lack the benefit of hindsight, the choices we make are often characterized by uncertainty over which choice is more likely to lead to a desired outcome or even which outcome might be preferable. This uncertainty is likely to increase when we have to make decisions for other people. In addition to the uncertainty over the decision itself, this adds the complication of getting inside someone else's head to make a decision which they would value. We have no direct access to other people's thoughts and feelings, but we can try and take their perspective, although this is a process often subject to an egocentric bias (Epley et al., 2004). How then do people go about predicting the value another person places on objects or experiences, and how accurate are those predictions? These surrogate utility estimates are likely to be extremely common and essential to a range of decisions from buying a birthday present of an appropriate value (Davis et al., 1986) to decisions about end-of-life care (Moorman and Carr, 2008). Despite this little is known about the psychological processes involved in surrogate decision-making. In this paper we explore an important part of any surrogate decision, namely the estimation of another person's subjective utility.

### Surrogate decision-making

The majority of research on surrogate decision-making has been conducted in a medical context, in part because of the significance of the decisions, and also because the recipients of the decisions are often incapable of making decisions for themselves or are legally unable to give informed consent. For example, a growing literature is concerned with the accuracy of end-of-life care decisions (e.g., Moorman et al., 2009). Typically, in these sorts of studies patients and their next of kin are asked if they or their partner would choose a life-sustaining intervention in the event of a hypothetical medical scenario that might prevent death but leave the patient with a poor quality of life. Accuracy is measured as the agreement rate between the next-of-kin's prediction about what their partner would choose, and what the patient actually chose (Fagerlin et al., 2001). In the United States and many other countries the optimal outcome is that the surrogate makes a judgment that meets the Substituted Judgment Standard (Stanley, 1989) which requires that the decision-maker set aside their own preferences, and make a decision that they believe the patient would choose if they were able to do so, even if this contradicts the surrogate's own beliefs. However, a recent systematic review of the literature concluded that on average the agreement rate between next-of-kin and patient is in the region of 66% (Shalowitz et al., 2006). What these studies all show is that people are above chance in their predictions but less than perfectly accurate, even when the surrogate and beneficiary are familiar with one another. Unfortunately, because the decisions are essentially binary in nature they tell us little about the underlying psychological processes that the surrogate might use to make those decisions and where they might fail. Surrogate decision-making must involve an assessment of the beneficiary's attitudes to risk and their subjective utility. In this sense, surrogate decision-making may not differ from prospect theory or any other general descriptive model of decision-making for that matter. Despite this there have been few attempts to describe how the processes involved in making decisions are utilized in making decisions on behalf of another person. To date these all appear to be derivatives of Prospect Theory (Kahneman and Tversky, 1979).

*Social Values Theory* (Stone and Allgaier, 2008; Stone et al., 2013) predicts that the social value placed on the outcome is the predominant factor in the surrogate decision. In this model the surrogate uses an essentially heuristic process of recommending the option with the most socially sanctioned or desirable outcome instead of making a utility estimation. In this sense the surrogate decision-maker makes what is essentially an egocentric decision that benefits their own reputation rather than what the other person might prefer or what might be best for them. This heuristic could be motivated by a genuine expectation that the recipient's wishes would be consistent with social norms about the decision, and this could be more likely in situations with uncertain outcomes such as a medical decision. This model predicts that surrogate decision makers will make more risk averse decisions in situations in which safety is considered socially desirable or valued by the recipient compared to decisions in a context in which risk might be more desirable (e.g., romantic decisions, gambling). By contrast, when the decision maker makes a decision for himself or herself, they are more likely to engage in an analytic decision-making process. In many ways this model is consistent with Kahneman's Systems model (Kahneman, 2011). This model might account for some relatively trivial surrogate-decision, and perhaps even some profound ones too. But the model as it is described deals with a relatively narrow set of risky decisions that are different from utility based decisions that we are concerned with here. It seems unlikely that every surrogate decision is simple matter of heuristic reputation management. For example, it is unlikely that the next of kin makes an end-of-life decision, on the basis of what other people might think of the decision rather than weighing up the quality of life of their partner. Similarly, a physician when asked to recommend a treatment makes the decision on the basis of the expected outcomes and risks. Indeed there is some evidence that physicians might make more analytic decisions on behalf of their patients and heuristic ones for themselves (Ubel et al., 2011; Garcia-Retamero and Galesic, 2012). So although the model may be relevant to some kinds of decisions these may be limited. Nonetheless, if a legally designated surrogate decision maker were to base their surrogate decision on a social values heuristic then their intention would fail to meet the substituted judgment standard. The social values model does not claim, however, to address surrogate medical decisions.

The *Egocentric Anchoring and Adjustment* model (Epley et al., 2004; Epley and Gilovich, 2006) does include an analytic process for both surrogate and self decision-making. In this model, when making decisions on behalf of other people, or predicting the decision that another person might make, people use their own preference as a reference point or anchor. For example, they might initially analyse the decision in terms of what they would choose, and in doing so, implicitly or otherwise, make a subjective utility estimation. The surrogates then make the assumption that people have similar preferences to them and that they are likely to have a similar anchor point, whilst simultaneously recognizing that different people have different perspectives and preferences. The consequence is that the decision-maker adjusts their estimation of the other person's subjective utility around their own anchor point to reflect what they know of the differences between them and the other person. The adjustments involve discrete changes away from the anchor point and a satisficing stopping rule is applied after each adjustment. If the decision maker believes that the adjustment reflects the other person's perspective then the process stops, if not then a further adjustment is made. The combination of an anchoring and adjustment process and a satisficing rule results in a general egocentric bias in surrogate decision-making.

However, although this model can account for some errors in self-other decision making it does not make a distinction between predicting what another person *would* choose (a surrogate decision) and what they *should* choose (a benevolent decision), or account for the possibility that a surrogate decision maker may simply choose a course of action in their own favor rather than one that they think is in the beneficiaries' best interest or what they would choose for themselves. Indeed the model does not account for the common problem of people making decisions that they know to be less than optimal. Moreover, neither the Social Values model nor the Anchoring and Adjustment model account for how well the surrogate and beneficiary know each other. It seems reasonable to assume that the closer the relationship between two people then the more likely they are to be aware of each other's goals and values, and what they might choose to do in hypothetical situations. The Somatic Distortion theory of Surrogate Decision Making (Tunney and Ziegler, under review) attempts to capture the potential conflicts between the different perspectives one might encounter when making a decision on behalf of another person. The model incorporates factors that can influence perspective taking such as the familiarity between decision-maker and recipient and individual differences in empathy. In doing so the model should predict when a surrogate decision might be accurate and when surrogate decision-making might fail.

The Somatic Distortion model assumes that when making a decision on behalf of another person, the decision maker compares the preferred outcome from a number of different perspectives. The decision maker considers what they would do if they were the beneficiary of the decision, they simulate what they believe the beneficiary would choose, and what they believe the best outcome for the beneficiary would be irrespective of their own wishes or the beneficiaries'. We refer to each perspective as *Projected, Simulated*, and *Benevolent*, respectively. Of course, the decision-maker might simply decide what they would prefer the outcome to be irrespective of either the beneficiaries' wishes, or indeed what they would chose if they were the beneficiary. In this egocentric scenario the decision maker does not attempt to make a surrogate decision and no theory of surrogate decision-making is required. In computing a decision from each perspective the decision maker makes an estimated subjective utility judgment. The subjective utility estimation is distorted as a function of the participants' knowledge or familiarity with the beneficiary. For example, one is unlikely to have a casual conversation with a passing acquaintance about the relative merits of euthanasia, and so one might assume a stranger's preferences on average conform to social norms. On the other hand, we are much more likely to discuss, or share, our social values with our family and our close friends. Given that what we would choose might be different from what we think another person might choose, and that both perspectives could be different again from what we believe the best choice (the benevolent choice) to be, the model includes a simple majority choice rule to decide what the actual surrogate decision would be. Of course, empathy is an individual difference and we expect that the extent to which a surrogate utility judgment matches that of another person will vary not only as a function of external factors such as familiarity, but also of internal factors such as one's ability to take another person's perspective. In addition to the familiarity between the surrogate decision maker and the recipient, the model also assumes that the ability to take another person's perspective (empathy) affects the relationship between surrogates' predictions and preferences of the recipient. Faro and Rottenstreich (2006) provide some tentative evidence to this effect. They showed that an established measure of empathy (Davis, 1983) predicted the accuracy of surrogate decisions about risky choices. Our model predicts that relatively more empathetic people are more likely to make a simulated judgment than relatively less empathetic people who are more likely to make either a projected or egocentric decision.

A number of studies have shown that people make more impulsive decisions for themselves than they would do on behalf of another person, or that they predict another person would make. For example, Ziegler and Tunney (2012) asked participants to choose between a series of smaller-sooner and larger-later financial rewards for themselves or for a variety of people who varied in psychological distance from them. The results suggested a negative linear relationship between impulsivity and distance in which decisions for complete strangers were the least impulsive.

When asked to make decisions about risk we tend to assume that other people are more risk seeking than ourselves for both positive and negative scenarios, and, as with delay-reward discounting the effect is moderated by the construal distance of the outcome (Hsee and Weber, 1997). This gives rise to an empathy gap in which the decision maker feels the visceral consequences of a decision less for strangers than for the people they know (Loewenstein, 2000). This effect occurs for both standard lotteries and even sealed bid auctions (Chakravarty et al., 2011) and has clear implications for the investment strategy of traders in financial markets. In other situations that involve decisions about risky behaviors, but do not necessarily require an explicit risk computation, people show the same pattern of making or recommending riskier decisions for other people than they would for themselves. For example, Beisswanger et al. (2003) found that participants were more likely to recommend that other people engaged in risky romantic scenarios (e.g., a blind date) than they would choose to do themselves. Interestingly the effect was larger for trivial decisions than for more profound ones (dates vs. marriage: see also Stone et al., 2013).

In the two experiments that follow we assess the extent to which people are able to predict someone else's utility judgments and test the predictions of the Somatic Distortion Theory of Surrogate Decision Making that familiarity and empathy affect those predictions.

## Experiment one

In Experiment one we sought to develop a methodology to measure how similar surrogate utility estimation is to another persons utility judgments. In particular we wanted to measure the extent to which a utility judgment made on behalf of another person was a projection of their own preferences, or an accurate reflection of their partners' preferences. In keeping with the established literature we recruited older adults in established relationships and asked them to state their own subjective utilities for lifestyle items (selling price) or for health benefits (time trade-offs) using materials developed by Chapman and Johnson (1995).

### Method

#### Materials

The materials were a set of 10 health state trade-offs and 10 lifestyle items developed by Chapman and Johnson (1995) See Appendix A. Each participant rated two different versions of each trade-off: one that referred to themselves and one that referred to their partner. For the health state items the participants were asked to state the number of years added to their lives required to be willing to give up a particular health benefit, and also estimate the number of years they thought that their partner would require for the same exchange. For the lifestyle items the participants stated their minimum selling price, and predicted what their partners' minimum selling price would be for a set of lifestyle items.

#### Participants

The participants were recruited from the Lincolnshire branch of the University of the Third Age, an international organization that promotes life-long learning. The participants were 44 older adults in long-term relationships. All the participants were in heterosexual relationships resulting in the sample being composed of 22 males and 22 females. Their average age was 59.09 years (*sd* = 8.72), the average length of relationship was 31.68 years (*sd* = 10.66).

#### Procedure

Participants were tested in their pairs on separate computers and were not permitted to communicate with each other during the task. On the computer screens the participants were presented with the 40 items shown in Appendix A. Of these half were commodity/lifestyle items, and half were health related items. Each participant in each dyad provided two utility estimates for each item. For the health items they made a time trade-off and a predicted time trade-off for the other member of the dyad, so the utility for health items is expressed in years ranging from 0 to 50 years. For the lifestyle items the utilities are expressed in US dollars ranging from $0 to $20,000. The task was self-paced, and the order in which the items were presented was fully randomized for each participant. This study was approved by the Ethics committee in the University of Lincoln School of Psychology and conforms to British Psychological Society's Code of Human Research Ethics.

### Results

Each participant in each dyad made a time trade-off for each health item and a predicted time trade-off for their partner. The participants said that they would exchange on average 12.49 years (*sd* = 8.29), and predicted that their partner would exchange 13.07 years (*sd* = 8.73). This difference was not significant (*t*_43_ < 1.0). The difference between predicted partners' trade-offs and their partners' actual trade-offs was also not significant (*t*_43_ < 1.0). The average selling price for lifestyle items was slightly higher for oneself ($1005.32, *sd* = 2235.74) than they predicted for their partners although this difference did not reach significance ($785.94, *sd* = 1699.05, *t*_43_ = 1.83, *se* = 119.96, *p* = 0.07). The difference between what the participants predicted their partners' selling price would be and their partners' actual selling price was not significant (*t*_43_ < 1.00).

For each participant we first computed the within-subject correlations between their own utility judgments and what they predicted for their partner. We refer to this correlation as *projection* as it is a measure of how closely one's own preferences are to the predictions about another person's preferences. The average correlation coefficient between each participant's time trade-off for health items and what they predicted for their partner was significant (*r* = 0.42, *sd* = 0.37, *t*_43_ = 7.57, *p* < 0.01). The correlation between participants' own selling prices for the lifestyle items and what they predicted for their partners was also significant (*r* = 0.45, *sd* = 0.399; *t*_43_ = 7.41, *p* < 0.01).

These results nicely demonstrate a general agreement between participants' own utilities and how much they think their partners might value the same items. However, this might merely be a form of projected utility estimation. To determine the relationship between those predictions we next computed the within-subject correlations between the predicted utilities and the actual stated utilities of the other participant in the dyad. We refer to this measure as *accuracy*, because it is a measure of the association between the predicted preference and the other person's actual preferences. The average within-dyad correlation between what participants predicted their partners' selling price for the lifestyle items would be and what their partner actually stated was also significant (*r* = 0.33, *sd* = 0.396; *t*_43_ = 5.58, *p* < 0.01), as was the within-dyad correlation between predicted time trade-offs and actual time trade-offs (*r* = 0.39, *sd* = 0.37, *t*_43_ = 7.094, *p* < 0.01).

To determine the extent to which participants' surrogate decisions were independent of their own projected preferences or a simulation of their partners' wishes we next computed partial-correlations between what they predicted for their partner and what their partner actually stated while controlling for their own utility judgments. The mean partial correlation coefficient for health items was reliably greater than chance (mean *r* = 0.26, *sd* = 0.41; *t*_43_ = 4.25, *p* < 0.01) and the mean partial correlation coefficient for lifestyle items was also reliably greater than chance (mean *r* = 0.25, *sd* = 0.42; *t*_43_ = 3.87, *p* < 0.01). This suggests that participants are able to predict their partners' utility judgments as being different to their own. We interpret this as evidence of simulation in surrogate utility estimation rather than simple projections.

These data confirm the view that participants' estimates of their partners' utility judgments are similar to their own. These data are consistent with both the Anchoring and Adjustment Model, but also the Somatic Distortion Model. Of course the observation that these predictions are relatively close also confirms the view that people place similar values on similar things. However, when we partialled out participants' own utility estimates the correlations between predicted utilities and actual utilities remained significant. Our interpretation of this is that participants are able to set aside their own subjective utilities in order to estimate their partners'. This would not occur if the overall correlations were due to an egocentric anchoring and adjustment. Of course, these participants are very familiar with one another and one might expect that they would be familiar with their partners' preferences.

Interestingly, we found a negative correlation between the length of relationship between partners and the partial coefficients between their predicted and actual judgments for commodity items (*r* = −0.394, *p* < 0.01). This indicated that familiarity is predictive of how close a surrogate utility judgment is to another person's actual preferences. But that the longer the participants' relationships were, then the less independent the predicted judgments were of their own utilities indicating that familiarity may lead to greater projection. We found no such relationship for health judgments (*r* = −0.211, *p* > 0.05). This may be because there is a greater variation in individual preferences for commodity items (we may not all value art), but we share similar preferences for good health.

## Experiment two

Experiment 1 demonstrated that people who are familiar with one another are good at predicting each other's preferences and utility estimates for both health and commodity items. However, it may be that people generally share similar preferences and utilities and that this agreement may be due not to familiarity with another person, but to common estimates of the value of items. In Experiment 2 we ask whether people who are relatively unfamiliar with each other, or who do not know each other at all are able to predict each other's utility judgments. We also test the straightforward prediction that the relationship between surrogate predictions of another person's preferences is dependent upon their ability to empathize with another person.

### Method

#### Participants

The participants were recruited from the undergraduate populations at the University of Lincoln and the University of Nottingham. The Nottingham sample was composed of 84 females and 16 males, the Lincoln sample of 15 males and 71 females. Their mean age was 22.61 years (*sd* = 2.60). The participants were allocated to random dyads. This resulted in a total of 93 dyads, of which 70 were same sex pairs. Less than half the participants (60) said that they knew the other participant in their pair.

#### Materials

The materials were identical to those used in Experiment 1 with the addition of the Davis (1983) Interpersonal Reactivity Index to measure perspective taking and empathy. This has four subscales that map onto common conceptions of empathy namely Perspective Taking, Fantasy Scale, Empathic Concern, and Personal Distress.

#### Procedure

The procedure was identical to that used in Experiment One with the exception that the assignment of participants to each dyad was random and the participants completed the Davis Interpersonal Reactivity Index after the utility judgments. The order of presentation for the utility judgments was fully randomized for each participant. This study was approved by the Ethics committees in both the University of Lincoln and The University of Nottingham Schools of Psychology and conforms to British Psychological Society's Code of Human Research Ethics.

### Results

The average number of years that participants said they would trade for the health items was 20.05 years (*sd* = 9.97) and predicted that the other participant in the dyad would trade on average 20.49 years (*sd* = 10.28). This difference was not significant (*t*_185_ = 1.17, *se* = 0.37, *p* = 0.24). However, the average selling price for lifestyle items was slightly higher for oneself ($1620.38, *sd* = 1885.61) than was predicted for other participants in the dyads ($1449.58, *sd* = 1716.28, *t*_185_ = 2.27, *se* = 75.18, *p* = 0.02).

As a measure of projection we first computed the within-subject correlations between their own utility judgments and what they predicted for their partner. The average projection correlation between health utilities for oneself and predicted for the other participant in each dyad was significant (mean *r* = 0.42, *sd* = 0.41, *t*_185_ = 13.86, *p* < 0.001). The correlation between lifestyle utilities for oneself and predicted for the other participant was also significant (*r*_186_ = 0.48, *sd* = 0.42, *t*_185_ = 15.78, *p* < 0.001), and comparable to the correlation for health utilities. These results nicely demonstrate a general agreement between our own utilities and how much we think other people might value the same items. However, this might merely be a form of projected utility estimation. To determine the relationship between those predictions we next computed the within-subject correlations between the predicted utilities and the actual stated utilities of the other participant in the dyad (i.e., accuracy). The average accuracy correlation between the predicted health utilities and the other participants' actual health utilities was lower but nonetheless significant (mean *r* = 0.29, *sd* = 0.35, *t*_185_ = 11.50, *p* < 0.001). The correlation between the predicted commodity utilities and the other participants' actual commodity utilities was again lower but also significant (mean *r* = 0.25, *sd* = 0.0.43, *t*_185_ = 7.74, *p* < 0.001).

The Somatic Distortion Theory predicts that people who know each other will be better at predicting another person's preference than those who do not. The average within-subject correlation coefficients between predictions and actual preferences are shown in Table 1. Fisher's (1915) transformations showed that the correlations between participants' own and predicted utilities (projection) and between predicted and actual utilities (accuracy) were significantly higher when the participants knew each other compared to when they did not (*t*_184_ = 2.92, *p* = 0.004; and *t*_184_ = 2.75, *p* = 0.006, respectively). There was no difference between familiar dyads in participants' own and predicted health utilities (*t*_184_ = 1.45, *p* = 0.15), but familiar dyads were more closer in their predictions of their partners' health utilities (*t*_184_ = 2.07, *p* = 0.04).

**Table 1:** **Within-subject correlation coefficients for projection and for accuracy for health and lifestyle items shown separately for familiar and unfamiliar dyads in Experiment 2**.

	**Projection**				**Accuracy**			
	**Health**		**Lifestyle**		**Health**		**Lifestyle**	
	***r***	***sd***	***r***	***sd***	***r***	***sd***	***r***	***sd***
Familiar	0.478	0.371	0.607	0.364	0.369	0.375	0.369	0.445
Unfamiliar	0.385	0.423	0.421	0.427	0.257	0.329	0.186	0.413

To what extent are participants able to estimate another person's subjective utilities independently of their own preferences? To measure surrogate accuracy independently of projection we computed the correlation between the utility judgment that they predicted their partner would make and their partners' actual utility judgment, controlling for their own stated utility. The mean partial correlation coefficient for health items was significantly greater than chance (mean *r* = 0.176, *sd* = 0.36, *t*_185_ = 6.60, *p* < 0.001). For lifestyle items mean partial correlation coefficient was also significant (mean *r* = 0.09, *sd* = 0.44, *t*_185_ = 2.80, *p* = 0.006). Fisher's (1915) transformation revealed these two coefficients were significantly different from each other (*t*_185_ = 2.13, *p* = 0.03). The difference in the correlations between lifestyle and health items might be due to the desire for good health being a shared value across participants and there being more variety in participants' preferences for lifestyle items.

#### Effects of empathy

The somatic distortion model predicts that the extent to which the predictions of the surrogate decision maker are accurate is determined by their ability to empathize with another person. What aspect of empathy enables a participant to make good prediction about another person's subjective utilities independently of their own? To determine this we entered the four subscales from the Davis IRI into separate multiple linear regressions to predict the correlation coefficients for health and commodity items separately for dyads who were familiar with one another and those who were unfamiliar. The regression models were not significant for the accuracy of the surrogate predictions (i.e., the correlations between predicted and other judgments). However, the regression models for the correlations between self and predicted others were significant (i.e., projection). The results shown in Table 2 indicate a different pattern of results for familiar and unfamiliar dyads. Empathic concern is positively associated with these projection measures (except unfamiliar dyads for lifestyle items) indicating that the higher empathic concern scores a surrogate has then the less likely they are to project their own preferences. For the lifestyle items there was a positive relationship between perspective taking and the correlation coefficients between judgments for self and predicted other indicating a degree of projection, but a negative relationship between empathic concern and the same judgments for self and predicted other indicating that the more empathic concern a person has then the less likely they are to project their own preferences. However, unfamiliar dyads showed no projection in the relationships between the IRI subscales and the correlation coefficients for the lifestyle items. There were more statistical associations between the IRI scales and the judgments made about health items. Familiar dyads showed a positive relationship between the fantasy scale scores and the projection correlation coefficients between judgments for self and predicted other, and as observed for the lifestyle items a negative relationship between empathic concern and the health judgments for self and predicted other. Unfamiliar dyads also showed a negative relationship between empathic concern and the health judgments for self and predicted other, and a positive relationship between personal distress and the health judgments for self and predicted other. Overall the most reliable predictor of the relationship between self and predicted preferences for other people (i.e., projection) is the empathic concern scale of the IRI. The negative nature of this relationship indicates that the greater participants scored on this scale then the less likely they were to project their own preferences onto another person (i.e., the weaker the association between the self-judgment and the predicted other judgment). In other words, a higher empathic concern is associated with less projection in surrogate decision-making.

**Table 2:** **Standardized regression coefficients for Davis Interpersonal Reactivity Indices as predictors of the correlation coefficients between self and predicted other surrogate utility judgments for familiar and unfamiliar dyads in Experiment 2**.

**Projection**	**Familiar**				**Unfamiliar**			
	***β***	***se***	***t***	***p***	***β***	***se***	***t***	***p***
**LIFESTYLE ITEMS**								
Perspective taking	0.31	0.01	2.11	0.04	0.09	0.01	0.92	0.36
Personal Distress	−0.14	0.01	−1.07	0.29	0.15	0.01	1.55	0.12
Empathic Concern	−0.30	0.02	−1.92	0.05	−0.18	0.01	−1.64	0.10
Fantasy	−0.05	0.01	−0.37	0.71	0.13	0.01	1.32	0.19
**HEALTH ITEMS**								
Perspective taking	0.16	0.01	1.15	0.25	0.10	0.01	1.01	0.32
Personal Distress	−0.08	0.01	−0.60	0.55	0.28	0.01	3.07	<0.01
Empathic Concern	−0.38	0.02	−2.43	0.02	−0.42	0.01	−4.03	<0.01
Fantasy	0.32	0.01	2.23	0.03	0.05	0.01	0.58	0.57

## Discussion

In two experiments we have shown that people are principally able to estimate another person's subjective utilities independently of their own preferences. This is true for older adults in long-term relationships, but also for younger adults who do not know each other well. In older adults we found that the length of people's relationships was related to the degree to which the surrogate projected their own preferences onto their partners preferences for lifestyle items. The ability of the younger adults in Experiment 2 to empathize with another person was predictive of their ability to discard their own perspective and estimate the other person's utility judgments.

The two experiments reported here provide a novel methodology for assessing the relative accuracy of surrogate utility estimation, and in doing so provide a useful means to assess the conditions under which surrogate decision-making might be relatively accurate and situations in which it might not. We have shown that familiarity is predictive of the accuracy of surrogate decision utility estimation for lifestyle items but not for health items. Similarly, we have shown that at least one component of empathy, namely empathic concern, is predictive of participants' ability to make their surrogate utility estimation independent of their own preferences.

Although these experiments are exploratory they nonetheless speak to the validity of the theories of surrogate decision-making described earlier. *Social Values Theory* cannot in the form described by Stone and Allgaier (2008) and Stone et al. (2013) account for our participants' ability to make utility judgments of the kind described here on behalf of other people. That is not to say that when asked to make surrogate decisions that the decision-maker might not take into account the social acceptability or otherwise of their decision, but we see no reason why surrogate decisions should be based on such a simple heuristic. Indeed surely significant decisions that we might make on behalf of other people will involve similar analytic processes as those made for oneself. The *Egocentric Anchoring and Adjustment* model (Epley et al., 2004; Epley and Gilovich, 2006) fares better in that it predicts that decisions for oneself and for other people involve analytic processing that can take the form of utility estimation. The model is limited however because it does not account for external (to the decision-making process) sources of error such as the familiarity of the surrogate and beneficiary, or internal sources of error such as the ability to set aside one's own perspective and simulate or adopt another person's preferences. Indeed this is precisely the result of the regressions reported in experiment two in which people who score higher on perspective taking are better able to separate their own utility judgment and their prediction for another person. The data reported here are consistent with the predictions made by the *Somatic Distortion Model* in which participants decide upon their own preferences before setting aside their own perspective and simulating what they think the other person might prefer. This model accounts for both internal and external sources of error such as familiarity with the recipient and the decision-makers' ability to empathize with other people.

## Concluding remarks

Although these experiments do not speak directly to surrogate decision making in a medical context they do demonstrate that under optimal conditions, when there is no stress attached to the decision, neither in terms of an evaluation of the surrogate decision by the recipient (as in receiving a gift) nor in terms of actually experiencing the consequences, people are capable of estimating someone else's values and judgments. Taking into account someone else's subjective utilities is a central foundation of making a best interest judgment and it is thus encouraging to see the high success rate.

### Conflict of interest statement

The authors declare that the research was conducted in the absence of any commercial or financial relationships that could be construed as a potential conflict of interest.
