# Supplementary material for: Surrogate utility estimation by long-term partners and unfamiliar dyads
Source: Front Psychol. 2015 Mar 25;6:315. doi: 10.3389/fpsyg.2015.00315 (PMC4373268; doi:10.3389/fpsyg.2015.00315)
Supplement: Supplementary file 1 [file DataSheet1.PDF]

Appendix A

Health items

1. Three pills which would definitely stop a headache (i.e. [you/your partner] could use it to stop any 3 headaches).

2. A new treatment that would give [you/your partner] 20/20 vision: [you/your partner] partner would never need glasses or contact lenses.

3. A healthy eating plan that would cause [you/your partner] to lose 10 pounds and keep them off for 10 years.

4. A medical treatment which would completely eliminate all health risks caused by eating foods high in fat or cholesterol.

5. Three doses of pain reliever that will completely eliminate all pain during dental procedures.

6. For 1 week your partner is in such tremendous physical condition that they could compete in a triathlon.

7. A preventative medical treatment which would eliminate any chance that [you/your partner] would develop any form of cancer for the rest of their life.

8. For 3 winters [you/your partner] is completely free of cold, flu, or asthma symptoms.

9. [you/your partner] partner suddenly develops near professional-level athletic ability for their favourite sport.

10. For 3 years [you/your partner] is completely free of insect bites, poison ivy and hay fever.

Lifestyle items

1. An original painting of [your/your partner's] favourite *coastal* landscape, painted by a talented but unknown local artist.

2. Five round-trip *train* fares, anywhere in the *UK.*

3. One ticket to see Les Miserables in the *London West End.*

4. Three coffee table books of photographs of famous *UK* sights.

5. One ticket to a concert of your favourite musical artist at *any UK venue*.

6. Two tickets to a *West End show* of your choice (transportation to *London* included)

7. One round-trip air fare anywhere in *Europe*.

8. Three days of vacation at a beach house in *Cornwall* (including transportation and expenses)

9. Four dinners at a local restaurant.
